# Wood mouse feeding effort and decision-making when encountering a restricted unknown food source

**DOI:** 10.1371/journal.pone.0212716

**Published:** 2019-06-19

**Authors:** Mª Carmen Hernández, Álvaro Navarro-Castilla, Isabel Barja

**Affiliations:** Departamento de Biología, Universidad Autónoma de Madrid, Campus Universitario de Cantoblanco, Madrid, Spain; Ben-Gurion University of the Negev, ISRAEL

## Abstract

Animals making foraging decisions must balance the energy gained, the time invested, and the influence of key environmental factors. In our work, we examined the effect of predation risk cues and experience on feeding efforts when a novel food resource was made available. To achieve this, we live-trapped wood mouse *Apodemus sylvaticus* in Monte de Valdelatas (Madrid), where 80 Sherman traps were set in four plots. Traps were subjected to two food-access difficulties in treatments consisting of three consecutive nights: open plastic bottles (easy) and closed bottles (difficult), both using corn as bait. To simulate predation risk, we set fox faeces in half of the traps in each plot. We also considered moonlight (medium/low) as an indirect predation risk cue. We analysed whether bottles had been bitten by mice and the gnawed area of each bottle was measured. Our results indicated that food access difficulty, experience, and predation risk determined mice feeding decisions and efforts. The ability of mice to adapt feeding effort when a new food source is available was demonstrated because a higher proportion of closed bottles exhibited bite marks and the gnawed area was bigger. Moreover, mouse experience was determinant in the use of this new resource since recaptured mice gnawed broader orifices in the bottles and the gnawed area increased each time an individual was recaptured. Additionally, direct predation risk cues prompted mice to bite the bottles whereas the effect of different moon phases varied among the food access treatments. This study provides direct evidence of formidable efficacy of wild mice to exploit a new nutrient resource while considering crucial environmental factors that shape the decision-making procedure.

## Introduction

According to the optimal foraging theory, choices made by animals when foraging and selecting food aim to maximise fitness [1, 2, 3]. The variable food availability challenges animals to evaluate the trade-offs between nutrient demands and the energetic cost of foraging, thereby selecting the type of food with the maximum net benefit [1, 2]. These changeable environmental conditions have led to the development of a wide array of adaptations to efficiently exploit and utilise heterogeneous food resources in several life forms [4, 5]. The mechanisms which underly feeding choices are rather diverse, with both endogenous and environmental factors involved in the decision process [6, 7]. It is known that animals possess the ability to learn about the characteristics of the items in their diet and that feeding choices are dependent upon experience [7, 8]. Moreover, gathering information about food availability in a novel environment comes at a cost, because exploratory behaviours increase exposure to predation and divert time and energy from other fitness-enhancing activities (i.e., foraging, reproduction, predation risk assessment, etc.) [9]. However, those individuals that early allocate more energy to acquire information can learn the true value of the environment more quickly, which can lead to higher fitness gains due to a more efficient exploitation [10]. In this manner, learning can provide animals with the key to quickly adapt to this ever-changing environment by displaying novel feeding strategies when new food sources are present.

On the other hand, there is convincing evidence of predation-risk influence on prey behaviour [11, 12, 13], complicating the decision-making process even more when it comes to feeding opportunities. Prey animals possess the ability to estimate predation risk and adjust their behaviour to reduce the probability of being eaten [14], which is critical in habitats where the magnitude of threat is spatially and temporally variable [15]. Chemosensory cues are of vital importance for predation risk assessment in mice [8, 16]; these chemical signals are crucial for prey species since signals can alert them to the presence of any potential predators and enable mice to procure information about their activity and diet [17], modulating daily activity patterns [13, 18] and feeding habits of prey [19]. Moreover, perceived predation risk can vary depending upon environmental factors such as habitat complexity and moonlight [13, 20]. The influence of moonlight on mammal behaviour and its relationship with predator-prey dynamics is well documented [21, 22]: for rodents, bright nights increase detectability by predators and thus, predation risk and consequently, rodent species tend to decrease their activity around full moon nights [12, 23]. Therefore, for prey species, feeding strategies should be a trade-off between predation risk avoidance and the benefits of obtaining energy [11, 12]; however, behaviours that maximise food intake often increase exposure to predation risk, so prey must gather environmental information, decide how to allocate resources, and then pursue the option which maximises their fitness [24]. Consequently, an animal’s ability to balance its energy budget should be an important selective force for the evolution of life-history traits.

The aim of this study was to analyse feeding efforts under a novel restricted food resource in the wood mouse (*Apodemus sylvaticus* Linnaeus, 1758). Particularly, we focused in testing the ability of mice to learn and develop new feeding strategies over a brief period of time. We also evaluated if feeding efforts performed under different food access restrictions were conditioned by direct or indirect cues of predation risk such as predator faeces and moonlight.

Regarding the hypotheses, we expected that mouse feeding effort and intake would be influenced by the difficulty of accessing food. Individuals would only spend energy trying to gain access to food if it was beneficial. H1: mice facing an easier food-access restriction treatment allocate less effort attempting to reach the bait than those facing a more difficult treatment.

Secondly, previous experience with the plastic bottles would be critical because it provides information to the mice about how to exploit this new food resource. H2: recaptured individuals interact more with plastic bottles and obtain more access to food than first-time captured individuals.

Thirdly, predator faeces would increase perceived predation risk; therefore, the mouse would decrease its activity to avoid being detected. H3: direct predator cues decrease mice interaction with food containers, feeding efforts, and thereby intake.

Lastly, moonlight can improve predator ability to detect prey, hence, mice would decrease activity during brighter nights to avoid being detected. H4: brighter nights decrease mouse interaction with food containers, feeding efforts, and intake.

## Materials and methods

### Ethics statement

This research complies with the regulations on the protection of animals used for scientific purposes (Directive 2010/63/EU of the European Parliament and of the Council of 22 September 2010 and the Spanish legislation (Royal Decree 53/2013). The study had the approval of the Autonomous Community of Madrid (reference number 10/240775.9.16) and favourable reports from the Ethics Committee of the Autonomous University of Madrid (CEI 73–1330).

### Study area

The research was conducted in Monte de Valdelatas near Madrid, Spain (40°32'15.0"N 3°40'55.6"W), a Mediterranean forest located at an altitude of 650 m a.s.l. The characteristic vegetation is holm oak (*Quercus ilex ballota*) and scrubland (gum rock roses *Cistus ladanifer*, thyme *Thymus zygis*, and umbel-flowered sun roses *Halimium umbellatum*). Wild predators occur frequently in this habitat, those of importance being the red fox (*Vulpes vulpes*) and the common genet (*Genetta genetta*) [11, 25].

### Live-trapping and data collection

Two trapping sessions were carried out in March 2017 and 2018 in four plots with similar vegetation and composition. The distance between plots was 35 m to ensure that they were independent and that they corresponded to different mice populations [8, 16]. In each plot, 20 Sherman live traps were set in a 4 x 5 grid with 7 m distance between them [8, 16]. Total trapping effort was 960 trap-nights (2 different trapping sessions x 4 plots x 20 traps in each plot x 2 food-access treatments x 3 nights per treatment). All traps were hidden under vegetation cover to protect animals from adverse weather conditions and bait was provided inside traps (see details below). Traps were opened at sunset and data collection was started after the sunrise daily.

All captured animals were identified to species by external morphology and each captured mouse was weighed with a scale (PESNET, 100 g, PESNET 60g). Sex and breeding condition were checked according to Gurnell and Flowerdew [26]. Sex was determined using the anal-genital distance, which is longer in males than in females. In breeding adult males, the testicles were bigger, whereas breeding adult females showed conspicuous nipples on the abdomen and thorax and the vaginal membrane appeared perforated. Harmless waterproof paints (Marking stick DFV, www.divasa-farmavic.com) were used to mark captured individuals in non-conspicuous areas (e.g., ears, toes and tail) for discriminating recaptures [27]. Finally, all captured animals were immediately released after handling in the same place of capture.

### Predation risk simulation

To simulate predation risk, we used red fox faeces since this species is known to be present in the study area [11, 25], being one of the most common small mammal predators [28, 29]. Furthermore, red fox faeces have been previously demonstrated to effectively elicit anti-predatory responses [11, 12, 16]. Fresh faeces used for the treatment were obtained from captive red foxes (one male and one female) on a carnivorous diet from the Centro de Naturaleza Opennature Cañada Real (Peralejo, Madrid). We considered as fresh faeces only those with a layer of mucus, an elevated level of hydration, and strong odour [30, 31], and all faecal samples were frozen at -20 °C until treatment preparation. Seasonal and individual factors are known to influence volatile compounds among individuals [32, 33] so, to guarantee homogeneity (providing a similar degree of predation risk in all the treated traps, and therefore) and avoiding possible result bias, all collected red fox faeces were carefully mixed.

In each plot, half of the traps were subjected to a predator odour treatment consisting of 2 g of fresh fox faeces. Within the 4 x 5 grids set in each plot, predator treatment was set on two non-consecutive rows (10 traps) while the other two rows (10 traps) acted as controls (i.e., without predator faecal cues), these distances between treatments have been previously validated to be sufficient to detect changes in mouse behaviour [11]. To avoid the influence of border effects due to treatment distribution, control and predator treatment rows were alternated in each plot. The faecal material was placed on one side near the trap entrance to avoid blocking the entry for rodents but close enough to act as a potential predation risk cue (i.e., 3 cm away, approximately). Predator treatment was replaced every day at sunset to guarantee odour effectiveness when mice are more active, i.e., 2–4 hours after dusk [34].

Regarding indirect predation risk cues, since mice are known to be more active when the moonlight is dim due to a reduced predation risk perception [12, 35], we avoided trapping during highly illuminated conditions (i.e., full moon phases and nights near full moon). Thus, live-trapping sessions were carried out under low (< 25%, new moon) and medium (25–54%, waxing/waning crescent) moonlight conditions. The first live-trapping session began on a waning moon followed by a new moon while the second trapping session started on a new moon and ended with a waxing moon. Moon percent illumination corresponding to each sampling night was downloaded from the AEMet website (National Meteorological Service, www.opendata.aemet.es).

### Food access experiments

All traps were subjected to two different consecutive food-access treatments in which food-access difficulty was experimentally manipulated using polyurethane plastic bottles of 6 cm length, 2.7 cm total diameter, and 2 cm aperture diameter, baited with 5 g of toasted corn within. The first treatment (first three nights) consisted of opened plastic bottles inside all traps while for the second treatment (next three consecutive nights) all traps were provided with baited closed bottles (we made ten 1 mm holes with a needle in order to allow the mouse to smell the bait).

After trapping sessions, plastic bottles from the experiments were analysed in the laboratory to determine mouse feeding effort. For each bottle, we firstly confirmed mouse handling through the presence or the absence of bite marks made by individuals. To quantify the feeding efforts, we measured the total area gnawed by each mouse (i.e., size of the orifice made in the bottle). For this, gnawed areas were precisely transferred to translucent paper sheets which were then scanned. To measure the gnawed area, we analysed the scanned sheets through the Adobe Photoshop CC software in a similar way to [36], selecting the target gnawed area with the magic wand tool and using the image analysis tool to measure the gnawed area size in pixels.

Finally, to determine the amount of food eaten by each individual, we collected the unconsumed bait from each trap. The remaining bait was dried at 80 °C in a heater for 1 h to eliminate moisture and weighed with an electronic balance (C-3000/0.01 g CS, COBOS; precision 0.01 g). Thus, food intake by each individual was obtained by deducting the remaining bait weight to the initial 5 g of corn supplied inside each bottle.

### Statistical analysis

Since model residuals were not normally distributed, behavioural responses were analysed using Generalized Linear Models (GLMs). The robust estimator (Huber/White/ sandwich estimator) was used to correct homogeneous variances criteria deviations. To analyse factors triggering mouse handling of plastic bottles, a binomial distribution logit link GLM was performed measuring the response variable which was deemed the presence or absence of bite marks on the plastic bottles. Furthermore, to assess feeding effort, we used a GLM with a normal distribution and identity link, being the response variable linked to the missing area gnawed by each mouse in each bottle, measured in pixels. We also employed a GLM with normal distribution and identity link to test variation in food intake. For all models, the explanatory variables considered were the same: food access (opened bottle/closed bottle), recapture (first capture/recapture), moonlight (new moon/waxing or waning crescent), predation risk (control/predator), reproductive status (breeding/non-breeding), and sex (female/male), including weight as a covariate. We also tested the interactions food access*recapture and food access*moonlight and we also conducted separate ANOVA tests to analyse whether the gnawed area varied through repeated consecutive recaptures. Finally, a nonparametric Spearman’ correlation analysis was performed to check the relationship between the effort made by the mouse to obtain the bait (gnawed area) and food intake. Because mice did not need to gnaw open bottles to obtain the bait provided and due to the statistically significant relationship between food access with the extension of the gnawed area by mice, we only considered data from closed bottles for this correlation analysis.

Results were considered significant at α < 0.05. Data are represented as mean ± standard error (SE). The software used to perform the statistical analysis was SPSS 23.0 for Windows (SPSS Inc, Chicago, IL, USA).

## Results

### Bite marks

The total number of captures was 142, corresponding to 84 different individuals. The results of the binomial model showed that food access, recapture, predation risk, and the interaction between food access and moonlight were the factors which best explained the presence of bite marks in bottles (Table 1). In open bottles (N = 89), only 33.7% showed bite marks whereas in the closed bottles (N = 53) 90.6% of them were bitten by the mice. 40.5% (N = 84) of the first-time captured mice bit the bottles while this percentage increased to 75.9% for recaptured ones (N = 58). For predation risk influence, we found bite marks in 67.5% (N = 51) of the bottles treated with fox faeces, which was lower in the absence of predator cues (50.0%, N = 27). Regarding the interaction between food access and moonlight, we found that mice bite marks were less frequently found in open bottles during new moon nights (27.8%, N = 20), while this was higher during waxing/waning crescent moon nights (58.8%, N = 10). In contrast, bite marks appeared in the majority of the closed bottles independently of the moon phase: new moon nights recorded 95.7% (N = 22) and 86.7% (N = 26) during waxing/waning crescent nights.

**Table 1:** Results of the binomial logit GLM analysing the effects of individual, environmental, and experimental factors on the absence or presence of bite marks made by the mice in the plastic bottles.

Factor	*F*	df	*p*
Food access	14.113	1	0.000
Recapture	7.618	1	0.006
Moonlight	1.772	1	0.183
Predation risk	5.945	1	0.015
Reproductive status	0.022	1	0.883
Sex	2.627	1	0.105
Weight	0.242	1	0.623
Food access*Recapture	0.049	1	0.826
Food access*Moonlight	4.017	1	0.045
Residual degrees of freedom		132	

### Feeding efforts

**Fig 1:**
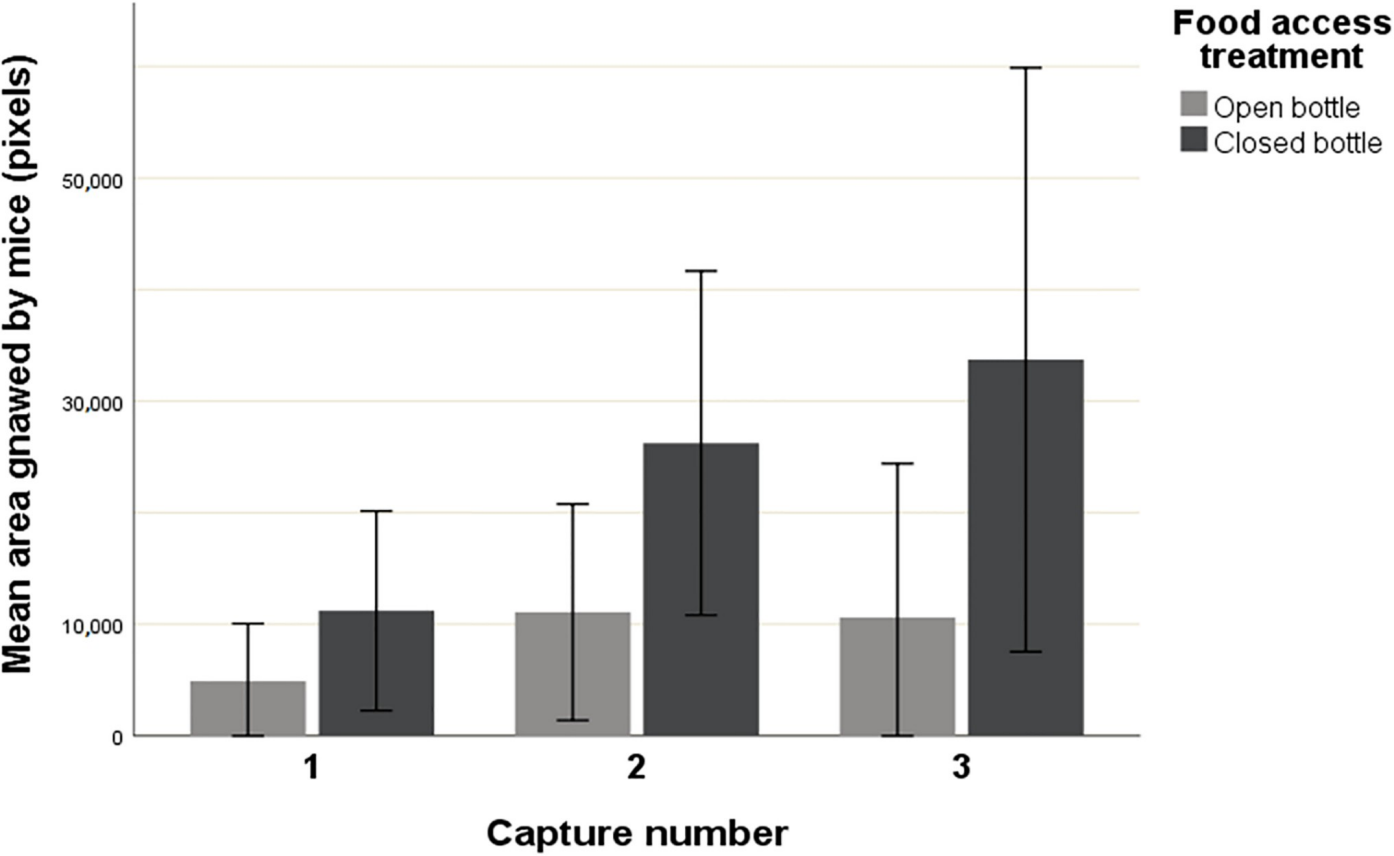
Mouse feeding effort (mean gnawed area ± SE) through consecutive captures of each individual depending upon the food access treatment (open bottle / closed bottle).

**Fig 2:**
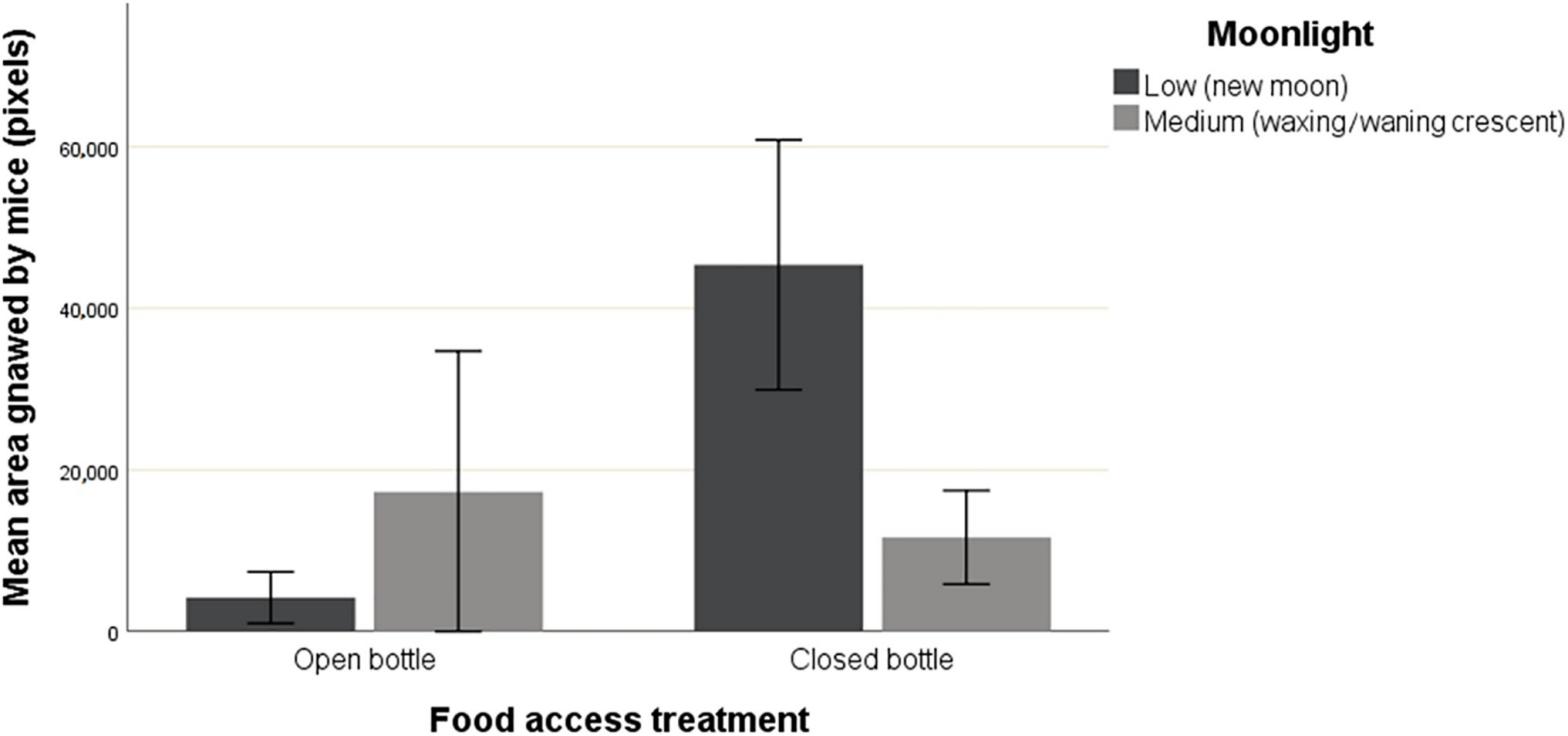
Mice feeding efforts (mean gnawed area ± SE) in relation to food access (open bottle or closed bottle) and the moonlight (low, new moon / medium, waxing-waning crescent).

Results of the GLM analysing mouse feeding effort (i.e., gnawed area) are shown in Table 2; the main influencing factors were food access, recapture, and moonlight. The average area gnawed by mice in open bottles was lower (6690.0 pixels ± 2141.0 SE; N = 89) than in closed ones (26277.4 ± 4361.0; N = 53). Overall, first-capture mice gnawed an average area of 6499.8 ± 2213.9 (N = 84), while a broader area was performed by recaptured individuals (24864.3 ± 4090.5 pixels N = 58). Interestingly, separate analyses showed that the area gnawed by mice increased exponentially during consecutive recaptures (*F*_4,48_ = 7.641, p < 0.001), but this significance was driven by individuals facing closed bottles (*F*_4,48_ = 3.226, p < 0.05; see Fig 1). Furthermore, the interaction between food access and moonlight showed that mice gnawed particularly broad areas of the closed bottles during new moon nights (45373.4 ± 7735.7; see Fig 2).

**Table 2:** Results of the GLM testing the effects of individual, environmental, and experimental factors on feeding effort (= area gnawed by mice).

Factor	*F*	df	*p*
Food access	4.811	1	0.028
Recapture	16.588	1	0.000
Moonlight	0.751	1	0.386
Predation risk	0.849	1	0.357
Reproductive status	1.556	1	0.212
Sex	0.626	1	0.429
Weight	0.015	1	0.902
Food access*Recapture	1.328	1	0.249
Food access*Moonlight	16.483	1	0.000
Residual degrees of freedom		132	

### Food intake

**Fig 3:**
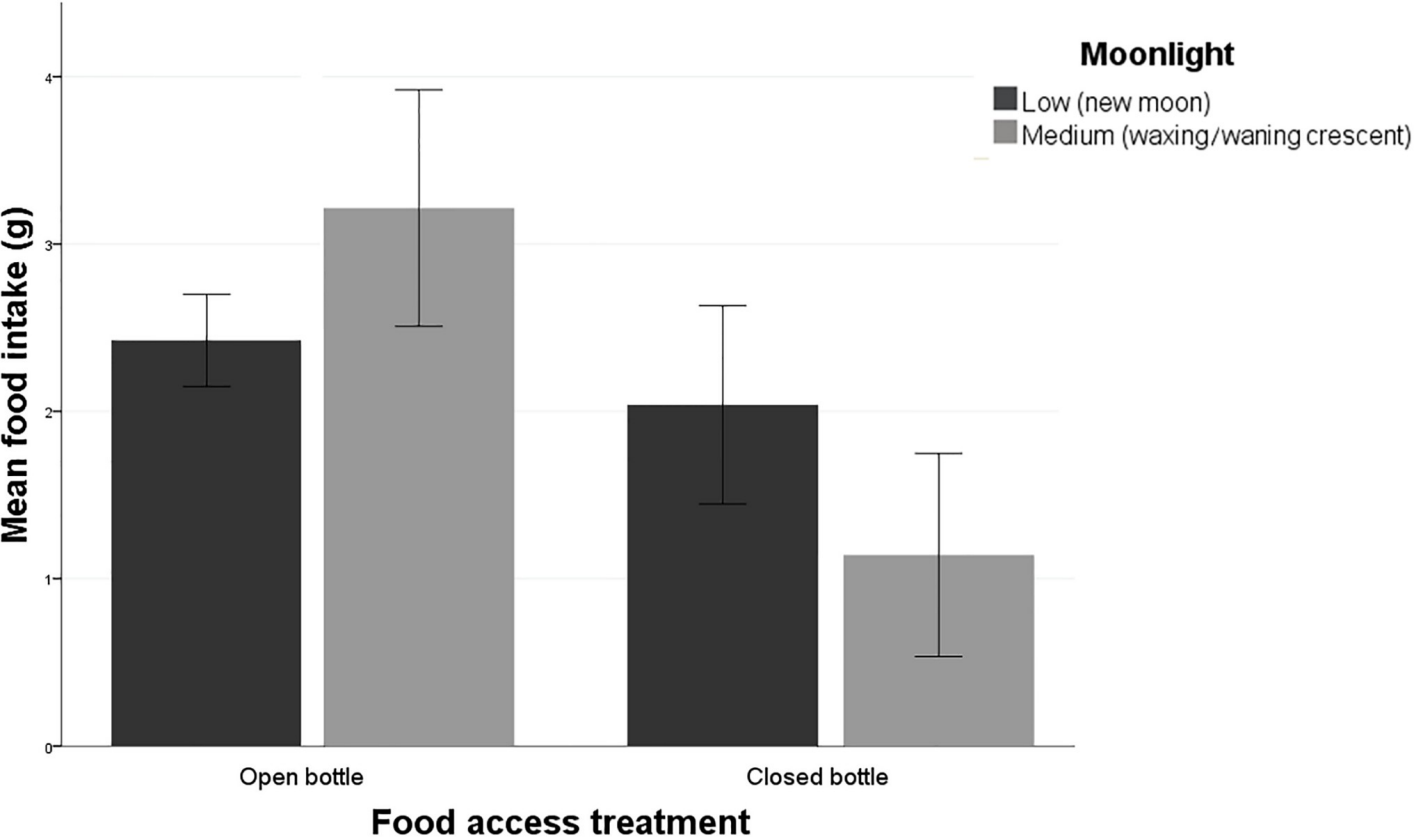
Mouse food intake (mean grams of corn consumed ± SE) depending upon food access (opened bottle or closed bottle) and the moonlight (low, new moon / medium, waxing-waning crescent).

Results of the GLM testing mice-food intake (Table 3) revealed that the main factors explaining it were food access treatment, recapture, and the interaction between food access treatments and moonlight. Individuals ate more in the open bottle treatments (2.6 g ± 0.1 SE; N = 89) than in the closed bottle ones (1.5 ± 0.2 g; N = 53). Additionally, first-time capture mice exhibited a decreased intake (1.9 ± 0.2 g; N = 84) compared to recaptured individuals (2.5 ± 0.2 g; N = 58). Moreover, the interaction between food access treatment and moonlight showed that mice intake was particularly low during medium moonlight nights when facing the closed bottle treatment (1.1 ± 0.3 g; N = 30; Fig 3) and especially high during medium moonlight nights in the open bottle treatment (3.2 ± 0.4 g; N = 17).

**Table 3:** Results of the GLM testing the effects of individual, environmental, and experimental factors on mouse food intake (g).

Factor	*F*	df	*p*
Food access	34.515	1	0.000
Recapture	20.351	1	0.000
Moonlight	1.231	1	0.267
Predation risk	0.011	1	0.917
Reproductive status	2.725	1	0.099
Sex	0.375	1	0.540
Weight	0.421	1	0.516
Food access*Recapture	1.717	1	0.190
Food access*Moonlight	6.593	1	0.010
Residual degrees of freedom		132	

Finally, a correlation analysis showed that there was a positive correlation between the effort made (i.e., the area gnawed) to obtain the bait and the intake of food (Spearman correlation, r = 0.805, N = 53, p < 0.0001).

## Discussion

### Bite marks

To our knowledge, this is the first study which provides evidence of the importance of experience and perceived predation risk in wood mouse feeding efforts and their decision-making process. As expected, food access difficulty determined the presence of bite marks in the bottles, indicating that the mice understood the implications of the feeding devices since they tended to spend extra energy on food handling only if it was mandatory (i.e., closed bottles). The presence of some bite marks and openings made by the mice in open bottles could be considered as exploratory behaviour to acquire information about the new food resource, because animals that learn faster about the value the environment can have higher fitness gains due to a more efficient exploitation [10]. Moreover, some biting and chewing not aimed to obtain food could have appeared as a stress-coping mechanism for being confined.

Experience also determined mouse choices in relation to effort, ‘to bite’ or ‘not to bite’, the food container. Naïve individuals were less inclined to gnaw the plastic bottles, demonstrating that experience is a decisive factor regulating wood mouse feeding choices when a new source of food is available [7]. Predator cues also affected the mouse decision-making process: in this case, fox chemical signals seem to have a stimulating effect which prompted individuals to interact with the food containers. Predator scents have been previously demonstrated to modify food intake [8, 11]; however, the direction of this association is not clear since there is evidence of both an increase and a decrease in the food intake. In our study, we hypothesise that traps could have provided the mice with a safe space to handle the food resources [8, 37]. As a consequence, these mice may have chosen to feed because they were sheltered against predator attacks. Alternatively, predation risk could have triggered physiological stress response in mice [16] and the immediate mobilisation of energy could have stimulated the mouse to bite the food containers.

Regarding the food access and moonlight interaction effect, while mice facing open bottles were more reluctant to try to get access to food during new moon nights, the moonlight did not influence mouse behaviour when the bottles were closed. When experiencing closed bottles, mice are compelled to bite the containers to obtain the food despite predation risk cues. In this particular setting, the prospect of obtaining a potentially highly nutritious food could counterbalance the risk of being detected [38, 39]. However, when biting the food containers is not required to accomplish feeding, individuals behave differently depending upon indirect predator cues. When moonlight was low, bite marks were less frequent on open bottles. We presume that gnawing the bottles can be noisy and thus, it can increase exposure to predators relying on auditory cues to detect prey [40]. This result seems to indicate that individuals were attempting to reduce the probability of being detected by eating the food through the opening in the bottle instead of gnawing it.

### Feeding efforts

In accordance with the previous results, food access difficulty determined the extent of mouse feeding endeavour, demonstrating that individuals optimally adjust their energy expenditure depending on food accessibility. Experience and learning have proved to be excellent adaptive features when it comes to feeding [10, 41], making individuals extremely resourceful and giving them the essential responses to survive in highly variable environments. Our study showed that experience prompted individuals to invest energy in attempting to gain food access and the skill of the procedure seems to be more efficient, since they managed to perforate a wider area of the bottles. On the other hand, we need to consider that a wider gnawed area could have been also explained by increasing feeding efforts and not only because of a skill improvement. In addition, the positive correlation found between the gnawed area and food intake confirmed that the endeavour they performed was justified, spending more energy only if they could counterbalance the feeding costs associated with that effort [42]. Our results indicate that mice are fast learners, improving their skill two-fold with only a single previous encounter with the food containers. However, this endeavour was only significantly improved in mice facing closed bottles, demonstrating again the ability of individuals to make efficient energy-budget decisions. The relevance of experience and learning upon mice feeding efforts is clear, providing mice the opportunity to exploit new food resources in a relatively short amount of time. Despite the fact that learning feeding techniques can have expensive associated costs in terms of energy and time [41], the highly variable natural living conditions could have induced the development of this remarkable evolutionary strategy by enhancing individual mouse fitness [11, 43].

Contrary to our predictions, predator faecal cues did not affect mouse feeding effort. Nevertheless, this result would be in accordance with other studies that discovered no effect of predator cues on feeding behaviour [12, 13]. As we suggested previously, traps could have been perceived as a refuge against predators, allowing them to feed in a secure environment [8, 37]. Another plausible explanation would be that due to individuals remaining several hours under the influence of predation cues, they must resume their feeding activity in order to not compromise their survival [43, 46].

As for the influence of the interaction between food access and moonlight on feeding effort, new moon nights were associated with increased feeding efforts when individuals were dealing with the more arduous treatment (i.e., closed bottles). This result gives us direct insight of mouse decision-making and the behavioural response elicited when a trade-off between predation risk and feeding is presented (see predation risk allocation hypothesis [43]). According to this theory, individuals would increase feeding effort during new moon phases when the perceived predation risk is low, since moonlight can increase prey detectability and hence, hunting success for predators [44, 45]. Thus, darker nights caused mice to feel safer, allowing individuals to spend energy in the device. On the contrary, a rise in perceived predation risk caused by the increase in moonlight probably caused mice to maintain a low profile and to choose survival over increasing their exposure for handling the food resource, even though the energetic reward was high. Furthermore, according to the optimal foraging theory, when predators are present, mice would stop feeding sooner because the marginal value of food relative to safety was lower. This result is in accordance with previous studies that show how mouse activity and food intake diminish with the increase in night luminosity [12, 23]. The variation found regarding this effect on the open vs. closed bottle treatments may be explained by the differences in handling efforts and the associated predation risks. In the open bottle treatment, mice can quietly feed through the opening. In contrast, mice are forced to chew the closed bottles to obtain the food, which it can be potentially noisy and requires increased locomotory activity. Consequently, mice may have felt safer in performing this task during low predation risk (i.e., darker) nights, while moonlight might have lost importance when food access was less complicated.

### Food intake

Wood mouse intake was modulated by food access restrictions and previous experience with the food containers. Mice decreased their food intake in closed bottles since the difficulty in obtaining food was particularly high. Also, it has been suggested that high handling costs can decrease food intake in unpredictable settings [47]; thus, individuals do not allocate too much energy in obtaining food if the prospect of acquiring it is not certain. For the recapture effect, it appears that previous experience with the food containers is crucial: when a new food resource appears, animals must gather information and assess the value of the new food before exploiting it [9, 10]. Therefore, the lower food intake found in first-time captured individuals could be explained by this phenomenon. Recaptured individuals had already learned the true value of the corn, they do not need to allocate time in assessing it, and thus, they can focus directly on feeding.

Although we expected a decrease in food intake in traps treated with fox faeces it appears that the presence of predator faeces does not affect food intake in this setting. As discussed above, the shelter provided by the traps against predators could have allow the mice to not interrupt foraging [8, 37]. Since mice remained trapped during the whole night, it may not be fitness-enhancing to stop feeding for such long periods of time [43, 46].

Regarding the interaction between food access and moon phases, it follows the same pattern as feeding efforts in the closed bottles. The higher predation risk perceived during medium moonlight nights caused a diminished food consumption due to the luminosity enhancing predator ability to detect the mice, so they chose to reduce their foraging effort because the marginal value of food in relation to safety may be considered lower. On the contrary, during the open bottle treatments, they did not need to chew the bottle to obtain the food, which would be potentially noisy, so mice did not decrease feeding because they could feed through the opening in the bottles.

Finally, we found that individual variables such as breeding condition, sex, and weight, had no effects on feeding behaviour. It may be possible that the higher energetic demands of certain individuals were only reflected upon the food intake rather than having an influence on the mice’s feeding efforts. Although this was not expected, the results clearly show that these factors were not determinant, and that experience and moonlight were the phenomena which modulated wood mice feeding choices and their efforts when a new source of food was made available. The wood mouse plays a key role in forest ecosystems, being a pivotal part of the diet of many often-endangered predators [48, 49]. These results provide certain hope about the resilience and plasticity of mice populations, frequently subjected to human-induced changes that can modify food resources and its availability.

## Supporting information

S1 Data Data collected and analysed in the present study. (SAV)
